# Catecholamines in Alzheimer's Disease: A Systematic Review and Meta-Analysis

**DOI:** 10.3389/fnagi.2020.00184

**Published:** 2020-09-11

**Authors:** Xiongfeng Pan, Atipatsa C. Kaminga, Peng Jia, Shi Wu Wen, Kwabena Acheampong, Aizhong Liu

**Affiliations:** ^1^Department of Epidemiology and Health Statistics, Xiangya School of Public Health, Central South University, Changsha, China; ^2^Department of Mathematics and Statistics, Mzuzu University, Mzuzu, Malawi; ^3^Department of Land Surveying and Geo-Informatics, The Hong Kong Polytechnic University, Hong Kong, China; ^4^International Initiative on Spatial Lifecourse Epidemiology (ISLE), Hong Kong, China; ^5^Faculty of Geo-Information Science and Earth Observation, University of Twente, Enschede, Netherlands; ^6^Department of Obstetrics and Gynaecology, University of Ottawa, Ottawa, ON, Canada; ^7^Ottawa Hospital Research Institute, Ottawa, ON, Canada; ^8^Department of Public, School of Postgraduate Studies, Adventist University of Africa, Nairobi, Kenya

**Keywords:** catecholamines, dopamine, epinephrine, norepinephrine, Alzheimer's disease, meta-analysis

## Abstract

**Background and Purpose:** Previous studies found inconsistent results regarding the relationship between Alzheimer's disease (AD) and catecholamines, such as dopamine (DA), norepinephrine (NE), and epinephrine (EPI). Therefore, the purpose of this study was to perform a systematic review and meta-analysis to evaluate the results of previous studies on this relationship.

**Method:** Literature retrieval of eligible studies was performed in four databases (Web of Science, PubMed, Embase, and PsycARTICLES). Standardized mean differences (SMDs) were calculated to assess differences in catecholamine concentrations between the AD groups and controls.

**Results:** Thirteen studies met the eligibility criteria. Compared with the controls, significant lower concentrations of NE (SMD = −1.10, 95% CI: −2.01 to −0.18, *p* = 0.019) and DA (SMD = −1.12, 95% CI: −1.88 to −0.37, *p* = 0.003) were observed in patients with AD. No difference was found in the concentrations of EPI between the two groups (SMD = −0.74, 95% CI: −1.85 to 0.37, *p* = 0.189).

**Conclusion:** Overall, these findings are in line with the hypothesis that reduced NE and DA may be an important indicator for AD (Registration number CRD42018112816).

## Introduction

Alzheimer's disease (AD) is characterized by attention deficits including apraxia, aphasia, agnosia, and progressive amnesia (Soldan et al., 2016). Its incidence has increased in recent years worldwide (Chibnik et al., 2017). Early screening focusing on AD biomarkers may not only be reliable and consistent but also help to implement timely interventions that could result in better prognosis and reduced disease burden (Fliessbach and Schneider, 2018).

Among the neurotransmitter abnormalities that have been investigated in AD, catecholamines, such as dopamine (DA), norepinephrine (NE), and epinephrine (EPI), have been intensively studied with regard to their roles in the neurotransmitter efflux that include mediating a number of cognition functions, working memory, recognition memory, and spatial memory (Yates et al., 1983; Liao et al., 2002; Nelson et al., 2011; Bensmann et al., 2019). Furthermore, recent neuropathological research found a link between molecular mechanisms, functional changes occurring in the catecholamines, and the neuropathology of AD (Youdim, 2018). Saldana et al. showed that dopaminergic cell death in the substantia nigra pars compacta (SNpc) was associated with the AD (Saldana et al., 2008). In addition, DA also plays a key role in both Amyloid beta formation and cognitive decline progression (Kumari et al., 2018).

Meanwhile, inconsistent results have been reported regarding the association of catecholamine concentrations with AD (Fitzgerald, 2010; Chalermpalanupap et al., 2013). However, some studies have demonstrated that DA concentrations were higher in AD patients than in controls (Yates et al., 1983). Existing data also suggested that NE and EPI concentrations may be elevated in some patients with AD (Fitzgerald, 2010). Although a systematic review on the pivotal role of DA and DA receptor in AD was published, subsequently there had been some similar studies that were published (Pan et al., 2019), suggesting that it was necessary to update the role of catecholamines in DA.

Furthermore, despite some qualitative literature reviews summarizing the association between the catecholamine concentrations and AD, they were unable to quantitatively estimate the magnitude and significance of the association and address several critical methodological issues of the published studies concerning this theory (Bharath and Andersen, 2004). Thus, there was need to reassess this association and address the related critical methodological issues in the published studies using meta-analysis, which is widely known as the gold-standard method for data aggregation. To date, no such method has been employed to address these questions.

## Methods

### Protocol Registration

This study used the Preferred Reporting Items for Systematic Review and Meta-Analysis (PRISMA) statement (Moher et al., 2009). Therefore, it was registered with the International Prospective Register of Systematic Reviews, PROSPERO, and the registration number is CRD42018112816.

### Search Strategy and Selection Criteria

English articles published before October 1, 2019, were searched in the electronic databases, Web of Science, PubMed, Embase, and PsycARTICLES, to identify relevant studies on the relationship between catecholamine concentrations and AD. Search terms were designed with help from experienced librarians as follows: TI (Alzheimer's Disease OR Alzheimer Syndrome OR Alzheimer Dementia OR Alzheimer OR AD) AND TX (Catecholamine OR Catecholamines OR Dopamine OR Dopamin OR Epinephrine OR Norepinephrine OR Noradrenaline OR Noradrenalin OR Adrenaline OR Adrenalin). Hand searching in the reference lists of eligible studies was conducted in parallel by XP and AK, and any disagreement between them was resolved by the corresponding author (AL).

### Eligibility Criteria

The eligibility criteria for inclusion of studies were specified as follows: (1) case–control studies or randomized controlled trials (RCTs), which investigated AD cases and healthy controls; (2) studies that used standardized diagnostic criteria for AD based on the Diagnostic and Statistical Manual of Mental Disorders (DSM) or other international standardized criteria; and (3) studies that reported mean and standard deviation (SD) of catecholamines. On the contrary, studies were excluded if they (1) were case reports or review articles; (2) studied AD in combination with other mental disorders, or in vascular dementia patients, who used drugs that had influence on the catecholamine concentration medications or psychotropic; (3) reported results of non-humans or vitro experiments; and (4) were gray literature (i.e., unpublished reports).

### Data Extraction

Data extraction was performed by two investigators (XP and PJ) independently. Their disagreements were resolved through discussion with the corresponding author (AL). Information on the following variables was extracted from each eligible article: first author's surname, year published, geographical location, mean and standard deviation of age, gender distribution, method of AD assessment, and catecholamine measurements comprising type of sample, assay methods, sample storage temperatures, and mean and SD of the catecholamine concentrations studied. The data were organized and saved in Excel and EpiData 3.0.

### Quality Assessment

The quality of the eligible studies was evaluated using the Newcastle-Ottawa Quality Assessment Scale (NOS) (Stang, 2010). This scale evaluates studies on three broad perspectives: (1) Selection, (2) Comparability, and (3) Outcome.

### Statistical Analysis

The concentrations of the catecholamines were compared between the AD patients and controls by observing the significance of standardized mean difference (SMD) and its corresponding two-sided 95% confidence interval (CI) (Higgins et al., 2003). The R software (version 3.5.1) was used to analyze these data. Heterogeneity between the eligible studies was quantified using the *I*^2^ statistic, whose significance was evaluated using the Cochran's *Q* statistic test. Specifically, maximal heterogeneity was indicated by *I*^2^ = 100%, while no heterogeneity was indicated by *I*^2^ = 0% (Higgins et al., 2009). Also, publication bias was assessed by a funnel plot and Egger's test, when the number of eligible studies under consideration was at least 10 (Egger et al., 1997). Besides, sensitivity analysis was performed by redoing the meta-analysis each time an eligible study was removed from the analysis (Pan et al., 2018). Finally, all the hypothesis tests were two-sided with 5% significance level.

## Results

### Literature Search

**Figure 1:**
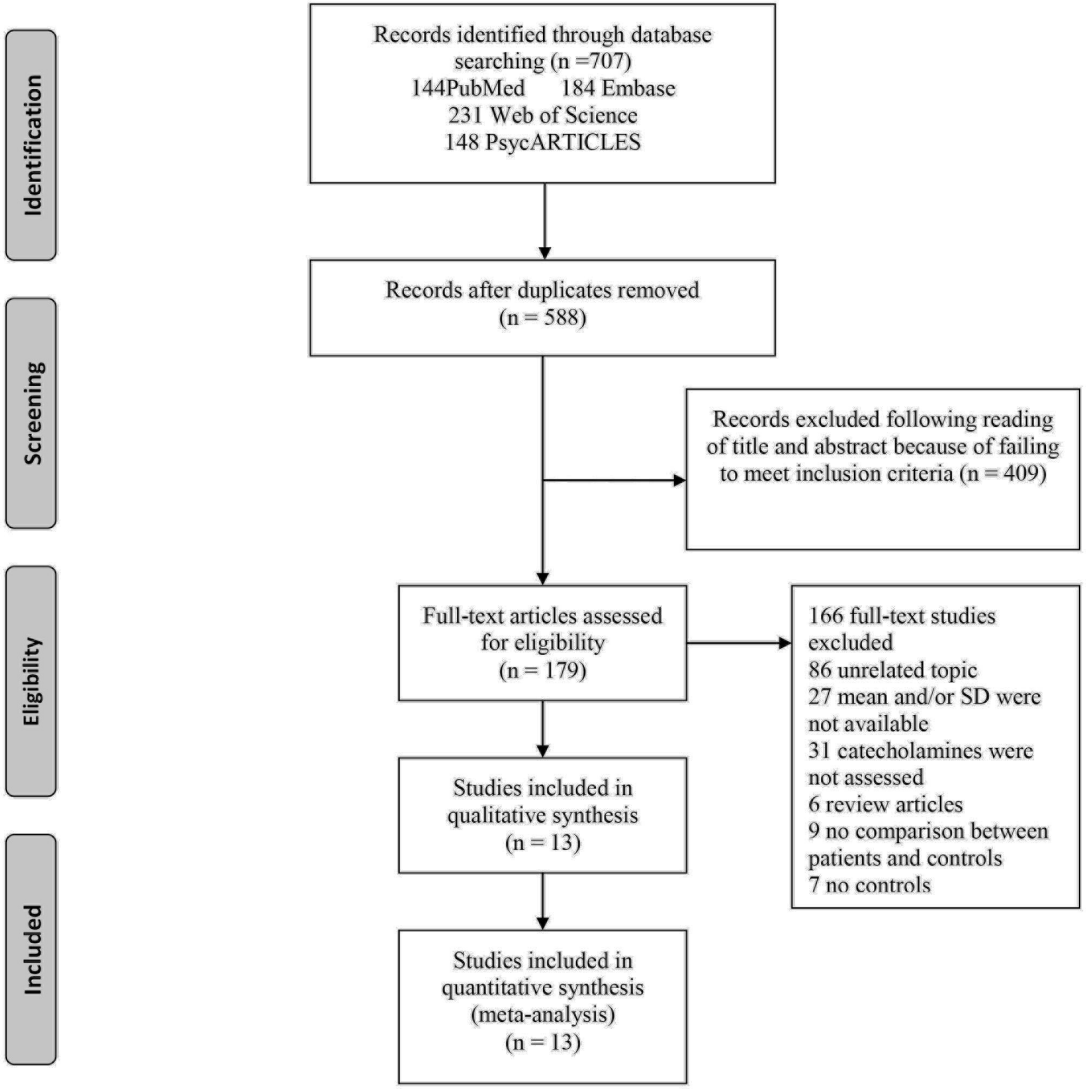
Flowchart of study selection. The process by which relevant studies retrieved from the databases were assessed and selected, or excluded is shown. Preferred reporting items for systematic reviews and meta-analyses (PRISMA) diagram for study search.

A total of 707 articles were identified, 184 from Embase, 231 from Web of Science, 144 from PubMed, and 148 from PsycARTICLES. Therefore, out of the 707 articles, 13 were considered as eligible for this study (Figure 1).

### Study Characteristics

Table 1 presents the characteristics of the 13 eligible studies. Accordingly, EPI concentrations were compared between 167 AD participants and 98 controls, while the concentrations of NE were compared between 219 AD participants and 213 controls, and the concentrations of DA were compared between 212 AD participants and 146 healthy controls.

**Table 1:** Characteristics of studies included in meta-analysis of catecholamines in Alzheimer's disease.

**Study**	**Material**	**Country**	**NOS**	**Female**	**Mean age**	**AD assessment**	**Collection time**	**Methods**	**Frozen**
Bemelmans et al. (2007)	Plasma	Netherlands	6	18 (86%)	85.2 ± 5.1	DSM-IV	AM 9:00	HPLC	−20°C
Elrod et al. (1997)	CSF	USA	6	18 (24%)	69.0 ± 6.0	DSM-IV	AM	RIA	−70°C
Kurup and Kurup (2003)	Plasma	India	5	0 (0%)	67.5 ± 5.7	ICD-10	NR	HPLC	NR
Lampe et al. (1989)	Plasma	USA	8	0 (0%)	64.3 ± 6.7	DSM-III	AM 9:00	RIA	−70°C
Liu et al. (2011)	Urine	China	5	0 (0%)	81.7 ± 3.8	DSM-IV	NR	HPLC	−20°C
Peskind et al. (1995)	CSF	USA	6	3 (30%)	69.5 ± 2.5	DSM-III	AM 9:00	HPLC	−70°C
Peskind et al. (1998)	CSF/Plasma	USA	7	18 (24%)	69.0 ± 6.0	DSM-III	AM 8:00	RIA	−70°C
Umegaki et al. (2000)	Plasma	Japan	7	66 (100%)	82.5 ± 7.8	DSM-IV	AM 7:00	HPLC	−70°C
Yates et al. (1983)	Brain	U.K.	6	4 (67%)	72.0 ± 18.0	Global dementia scale	NR	Other	−70°C
Adolfsson et al. (1979)	Brain	USA	7	13 (78%)	81.3 ± 7.2	Global dementia scale	NR	Other	−20°C
Allard et al. (1990)	Brain	Sweden	8	0 (0%)	82.0 ± 6.0	DSM-III	NR	HPLC	−70°C
Dekker et al. (2018)	Brain	The Netherlands	7	8 (73%)	81.3 ± 7.6	DSM-IV	NR	HPLC	−80°C
Snowden et al. (2019)	Brain	U.K.	7	7 (50%)	87.9 ± 8.9	DSM-III	NR	LC-MS/MS	NR

*Characteristics of eligible studies are shown in each independent case. AD, Alzheimer's disease; NR, not report; USA, United States of America; DSM, Diagnostic, and Statistical Manual of Mental Disorders; CSF, cerebrospinal fluid; RIA, radioimmunoassay; HPLC, High-performance liquid chromatography–tandem mass spectrometry; AM, Morning; NOS, Newcastle-Ottawa Quality Assessment Scale; U.K., the United Kingdom; LC-MS/MS, liquid chromatography–tandem mass spectrometry; ICD, International Classification of Diseases*.

### Overall Comparison

**Figure 2:**
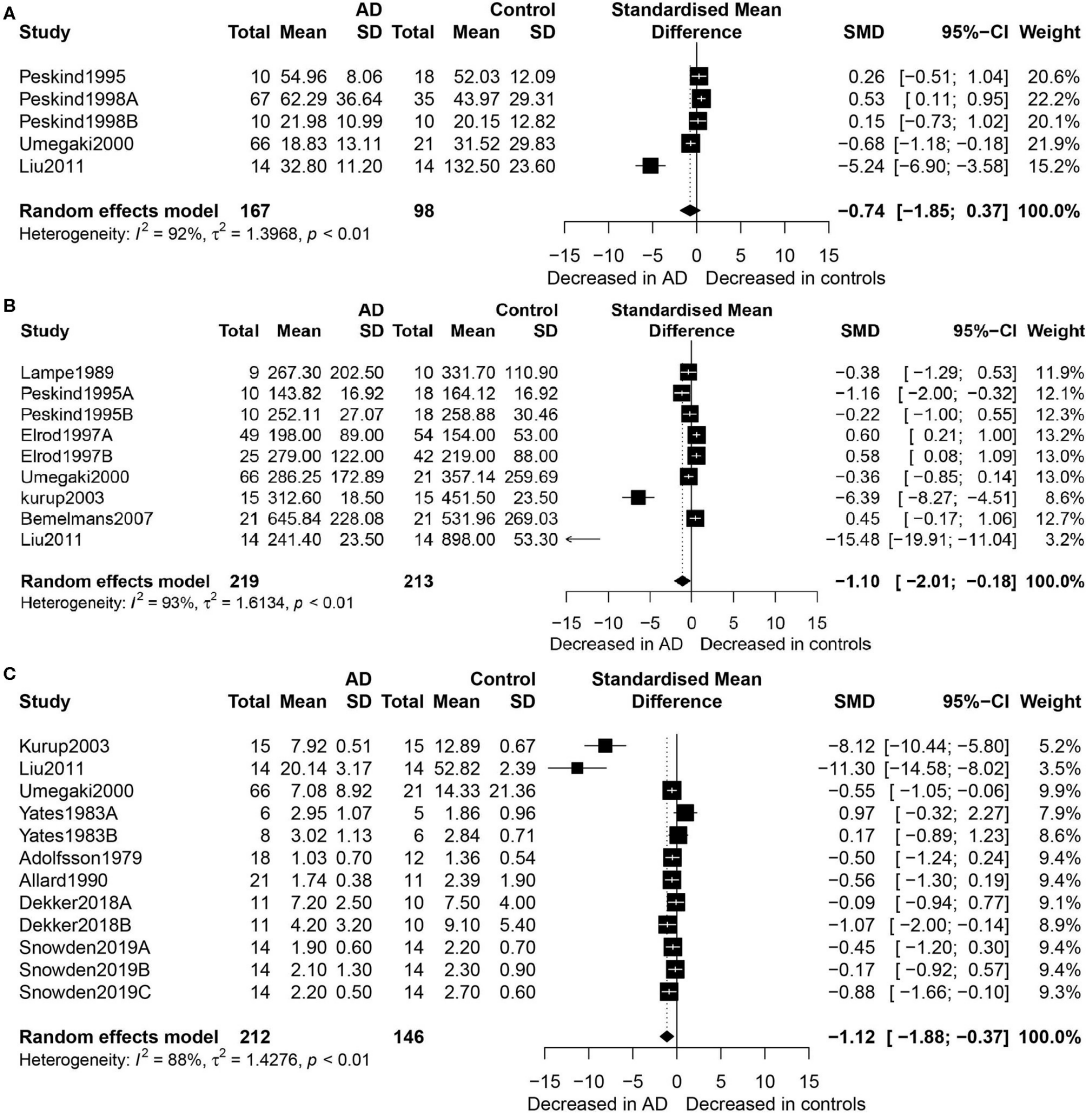
Forest plot of epinephrine **(A)**, norepinephrine **(B)**, and dopamine **(C)** between AD participants and controls. Study effect sizes of catecholamine differences between AD and controls. Each data marker represents a study, and the size of the data marker is proportional to the total number of individuals in that study. The summary effect size for each catecholamine is denoted by a diamond. AD, Alzheimer's disease; DA, dopamine; NE, norepinephrine; EPI, epinephrine; SMD, standardized mean difference.

Figure 2 shows the results of meta-analysis by the random-effects model. Thus, concentrations of EPI did not differ between the AD patients and healthy controls (SMD = −0.74, 95% CI: −1.85 to 0.37, *p* = 0.189) (Figure 2A). However, there were significantly lower concentrations of NE in the participants with AD than in the healthy controls (SMD = −1.10, 95% CI: −2.01 to −0.18, *p* = 0.019), and heterogeneity was considerable (*I*^2^ = 93.20%, Figure 2B). Regarding the eight studies that compared DA concentration levels between AD patients and healthy controls, significantly lower concentrations of DA were found in the AD patients than in the controls (SMD = −1.12, 95% CI: −1.88 to −0.37, *p* = 0.003), but with considerable heterogeneity (*I*^2^ = 88.20%, Figure 2C).

### Subgroup Analyses

Table 2 shows the results of subgroup analyses. Although EPI concentrations did not generally differ between the AD patients and healthy controls, they were significantly higher in AD patients than in the controls for studies conducted in the United States (SMD = 0.42, 95% CI: 0.08 to 0.76, *p* = 0.014), and with no heterogeneity detected (*I*^2^ = 0%). Similarly, when EPI was measured by the method of radioimmunoassay (RIA), there were significantly higher levels of EPI in patients with AD than in the healthy controls (SMD = 0.46, 95% CI: 0.08 to 0.84, *p* = 0.016).

**Table 2:** Subgroup analysis of dopamine, epinephrine, and norepinephrine between AD participants and controls.

	**Number of studies**	**SMD (95% CI)**	***Z***	***P*-value**	**Heterogeneity**		
					***Q* statistic (DF; *P* value)**	**τ^2^**	***I*^2^**
**EPI**							
All	5	−0.74 [−1.85; 0.37]	−1.31	0.189	52.11 4 <0.0001	1.4	92.30%
**Study country**							
USA	3	0.42 [0.08; 0.76]	2.45	0.014	0.80 2 0.6708	0.0	0.00%
Not USA	2	−2.89 [−7.36; 1.58]	−1.27	0.205	26.68 1 <0.0001	10.0	96.30%
**NOS**							
High	3	−0.01 [−0.85; 0.84]	−0.01	0.991	13.33 2 0.0013	0.5	85.00%
Other	2	−2.44 [−7.83; 2.95]	−0.89	0.375	34.78 1 <0.0001	14.7	97.10%
**Assayed methods**							
Other	3	−1.70 [−3.74; 0.34]	−1.63	0.103	34.79 2 <0.0001	3.0	94.30%
RIA	2	0.46 [0.08; 0.84]	2.40	0.016	0.60 1 0.4399	0.0	0.00%
**Gender**							
Female	4	0.06 [−0.59; 0.70]	0.17	0.867	13.57 3 0.0035	0.32	77.90%
Male	1	−5.24 [−6.90; −3.58]	−6.20	<0.0001	–	–	–
**NE**							
All	9	−1.10 [−2.01; −0.18]	−2.36	0.019	117.68 8 <0.0001	1.6	93.20%
**Mean age**							
>80	3	−2.73 [−5.20; −0.25]	−2.16	0.031	50.20 2 <0.0001	3.8	96.00%
≤80	6	−0.84 [−1.89; 0.21]	−1.57	0.116	66.08 5 <0.0001	1.5	92.40%
**Assayed methods**							
HPLC	6	−2.41 [−3.97; −0.86]	−3.04	0.002	94.03 5 <0.0001	3.2	94.70%
Other	3	0.41 [−0.04; 0.87]	1.80	0.073	3.97 2 0.1373	0.1	49.60%
**Gender**							
Female	3	−7.05 [−13.64; −0.46]	−2.10	0.036	68.60 2 <0.0001	31.94	97.10%
Male	6	0.04 [−0.46; 0.54]	0.16	0.873	22.98 5 0.0003	0.29	78.20%
**DA**							
All	12	−1.12 [−1.88; −0.37]	−2.93	0.003	93.50 11 <0.0001	1.4	88.20%
**Material**							
Brain	9	−0.38 [−0.70; −0.07]	−2.38	0.017	10.06 8 0.2612	0.0	20.40%
Other	3	−6.53 [−13.53;0.47]	−1.83	0.067	77.06 2 <0.0001	36.9	97.40%
**Gender**							
Female	9	−0.40 [−0.70; −0.10]	−2.61	0.009	10.23 8 0.2489	0.0	21.80%
Male	3	−6.53 [−13.47; 0.40]	−1.85	0.065	71.53 2 <0.0001	36.2	97.20%
**Assayed methods**							
HPLC	6	−2.77 [−4.36; −1.18]	−3.41	0.001	82.06 5 <0.0001	3.3	93.90%
Other	6	−0.28 [−0.70; 0.13]	−1.34	0.181	7.14 5 0.2104	0.1	30.00%

*Subgroup analyses are performed to compare the concentration of each catecholamine between the AD and the controls. Heterogeneity was quantified using I^2^ and its significance was tested using the Q statistics. DA, dopamine; AD, Alzheimer's disease; NE, norepinephrine; EPI, epinephrine; SMD, standardized mean difference; DF, degrees of Freedom; NR, not report; USA, United States of America; DSM, Diagnostic and Statistical Manual of Mental Disorders; RIA, radioimmunoassay; HPLC, High-performance liquid chromatography–tandem mass spectrometry; AM, Morning; NOS, Newcastle-Ottawa Quality Assessment Scale; U.K., the United Kingdom*.

In addition, lower NE concentrations were found in the AD participants than in the controls (SMD = −2.73, 95% CI: −5.20 to −0.25, *p* = 0.031) with respect to the over 80-year-old group, but these concentrations did not differ significantly between the two groups among the under the age of 80 group. Also, there were significantly lower concentrations of NE assayed by HPLC in the AD patients than in the controls (SMD = −2.41, 95% CI: −3.97 to −0.86, *p* = 0.002), but these concentrations did not differ significantly between the two groups with respect to the other assayed methods.

The results of sensitivity analysis indicated that there was no obvious influence of a single study on the outcomes of interest. Publication bias was not performed because the number of studies was <10 for each comparison.

Further, there were significant lower EPI concentrations in the male AD patients than in the healthy male controls (SMD = −5.24, 95% CI: −6.90 to −3.58, *p* < 0.0001), but these concentrations did not differ significantly between the two groups among the female participants. Conversely, there were significant lower concentrations of NE in the female AD patients than in the healthy female controls (SMD = −7.05, 95% CI: −13.64 to −0.46, *p* = 0.036), but these concentrations did not differ significantly between the two groups among the male participants. Similarly, the female AD patients had significantly lower DA concentrations than the healthy female controls (SMD = −0.40, 95% CI: −0.70 to −0.10, *p* = 0.009), but these concentrations did not differ significantly between the two groups among the male participants.

## Discussion

Generally, this study found that DA and NE concentrations were lower in the AD patients than in the healthy controls, indicating that DA and NE may have an important role in the AD. However, the findings suggested that there was no significant association between EPI concentrations and AD, implying that more studies are needed to confirm this outcome. As the population ages worldwide, AD is increasingly becoming a serious problem and hence warrants the need to explore modifiable risk factors, which would help to find effective therapeutic interventions to reduce the prevalence of AD (Papp et al., 2017). Elderly people with lower DA and NE might require more intensive interventions to reduce risk of AD. Thus, these findings are consistent with the catecholaminergic system dysfunction in the pathophysiology of AD, which has been well documented. For example, some studies reported that dopaminergic system dysfunction could be detected in AD patients at early stages of the disease, even in the absence of AD signs, such as extrapyramidal signs (Albin et al., 2013, 2015). Also, according to the amyloid plaque hypothesis of AD, Amyloid beta is neurotoxic and leads to the formation of neurofibrillary tangles and neuronal cell death in different regions of the brain, such as the cortical and hippocampus (Gordon et al., 2016). However, experimental studies have demonstrated that DA and its precursor L-dopa (L-3,4-dihydroxyphenylalanine) not only inhibited fibrillation but also appeared to dissolve existing Amyloid beta aggregates (Giunta et al., 2000).

**Figure 3:**
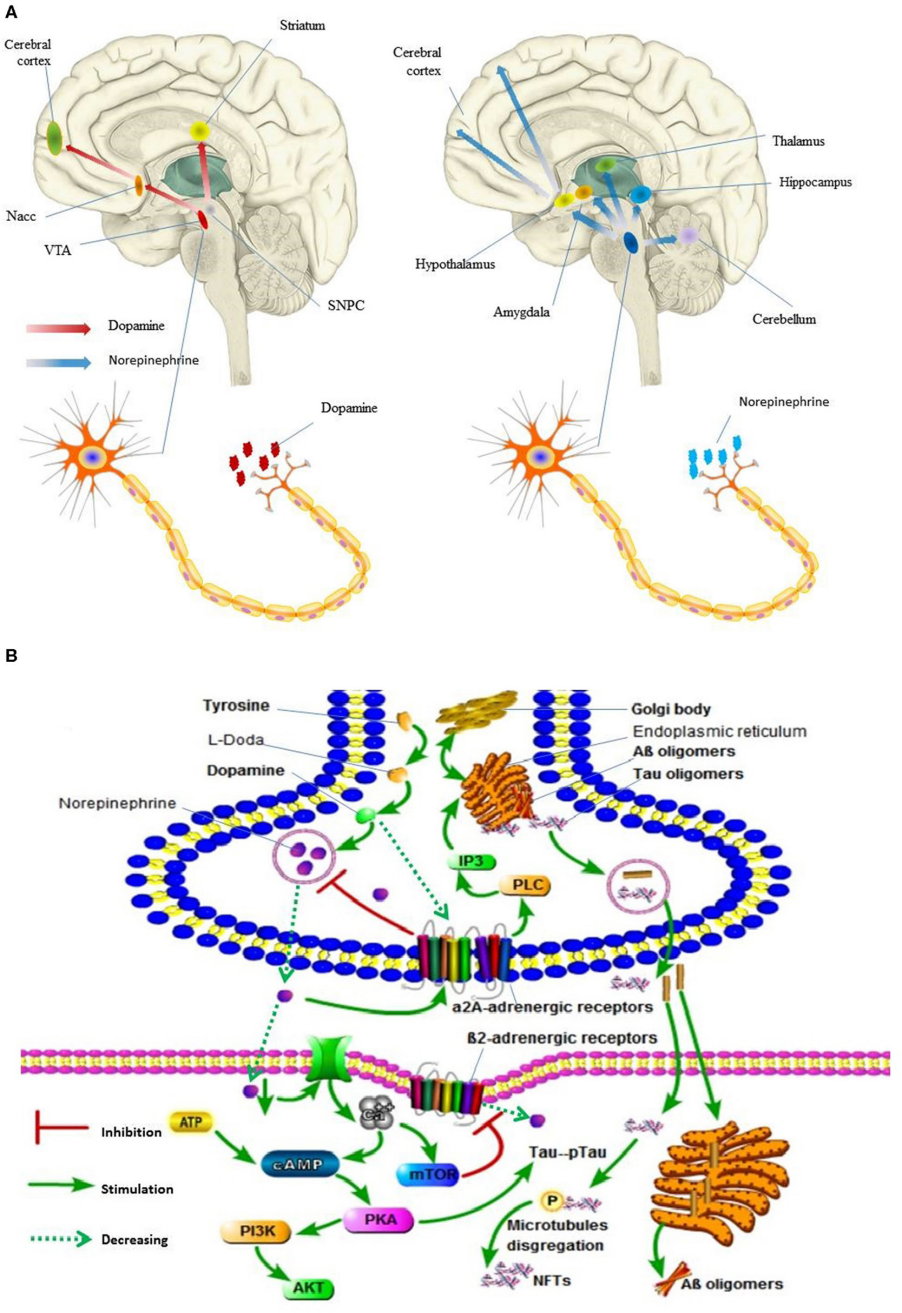
Dopamine and norepinephrine flow in human brain and its molecular events in synaptic. **(A)** Digital illustration of direction of dopamine and norepinephrine flow in human brain. Ventral tegmental area, VTA; accumbens nucleus, Nacc; Substantia nigra pars compacta neurons, SNpc. **(B)** Molecular events occurring in Alzheimer's disease following locus coeruleus dopamine and norepinephrine loss and autophagy impairment. pTau, PKA hyper-phosphorylates Tau protein; Neurofibrillary tangles, NFTs. In **(A)**, the brain and nerve cells are derived from the network, without copyright. In **(B)**, all the pictures are hand-drawn by us without any copyright. In this figure is drawn by AK.

As regards NE and EPI, previous studies indicated that they were associated with AD. This association could be explained by the fact that the ventral tegmental area (VTA) dopaminergic neurons mainly project to the accumbens nucleus (Nacc), amygdala, and the cerebral cortex, while the substantia nigra pars compacta neurons (SNpc) mainly project to the dorsal striatum (Figure 3A) (Krashia et al., 2017). Thus, several studies have suggested that tau lesions in the primary source of subcortical NE and EPI, and locus coeruleus (LC), may be the identifiable pathogenesis of AD (Zarow et al., 2003). Further, LC cells secrete the neurotransmitter NE in the brain, and the NE secreted by LC has important effects on the following regions of the brain: motor activity (cerebellum), spatial attention (prefrontal and parietal cortices, thalamus, and amygdala), learning and memory (hippocampus, prefrontal cortex, and amygdala), and arousal (cerebral cortex, thalamus, and hypothalamus; Figure 3A; Zarow et al., 2003). Moreover, several studies reported that tyrosine increases the prefrontal cortex dopamine and norepinephrine levels (Jaskiw et al., 2006). Considering oxidative stress, reduced concentrations of NE and IP3 contribute to the endoplasmic reticulum stress and impairment of proteins processing (Andres-Benito et al., 2017; Anton-Fernandez et al., 2017). PKA hyper-phosphorylates Tau protein, which leads to microtubule disaggregation and the formation of neurofibrillary tangles (NFTs) (Figure 3B; Kennedy et al., 2004).

In addition, other studies indicated that rotigotine, a DA receptor agonist, induces changes in both cortical excitability (increased) and central cholinergic transmission (restored) in AD patients (Martorana et al., 2013). Furthermore, the results of recent basic research supported the hypothesis that reduction of NE is associated with the pathophysiology of AD (Vermeiren et al., 2016). In particular, it was noted that cognitive impairment coincided with the LC atrophy, LC degeneration, and NE loss, and these have been considered as early stage signs of AD pathogenesis (Daulatzai, 2016). Furthermore, there is growing evidence suggesting that, while the physical process of LC neuron degeneration exacerbates AD-like neuropathology, the reduced NE impairs synaptic plasticity and cognitive performance (Rajkumar et al., 2017). Moreover, the inflammatory response induced and augmented by reduction of NE has been a key mechanism contributing to the initiation and progression of AD pathogenesis, microglia, endothelia, and astrocytes, which are among the major targets of inflammatory response (Bardou et al., 2014; Choi et al., 2015).

In general, NE is an anti-inflammatory molecule, and the deleterious effect of NE degeneration on the neuro-inflammatory response is the dysfunction of cellular machinery involved in Amyloid beta plaque metabolism and clearance (Lodeiro et al., 2017). Also in some animal studies, atomoxetine, a norepinephrine transporter inhibitor, demonstrated that treatment of 5XFAD transgenic mice (with Amyloid beta burden) elevated brain NE concentrations, increased expression of Amyloid beta clearance enzymes, reduced inflammatory changes, and improved spatial memory (Kalinin et al., 2012). Other clinical studies also suggested that atomoxetine was associated with improvement of cognition (Griffiths et al., 2018).

Although EPI was not associated with AD generally, there could be other moderators involving EPI that could be associated with AD. Therefore, the EPI concentrations in AD were considered for subgroup analyses just as the concentrations of DA and NE in AD (Dwibedi et al., 2018). Thus, according to the results of subgroup analysis, the association between EPI and AD was significant for studies conducted in the USA, unlike for studies conducted in the other countries. Nonetheless, EPI and NE results were inconsistent, probably because compensatory mechanisms may produce increased release of these neurotransmitters after loss of LC cells in AD (Fitzgerald, 2010).

However, it should also be noted that outlier values existed in this meta-analysis. For example, the forest map shows that the studies by Liu et al. and Kurup et al. may be outliers. There are several possible reasons for this. Firstly, these studies were conducted in China and India, respectively, whereas most of the other studies were conducted in Europe or America; hence, there may be differences due to population characteristics and ethnicity. Secondly, the quality score of each suspected outlier was lower (5 points) than that of the other studies in this meta-analysis.

Similarly, significantly higher concentrations of EPI were found in the AD patients than in the controls when EPI was assayed by RIA, whereas no significant differences in the same were observed between patients with AD and controls when it was assayed by other methods. These results may suggest that RIA could be a more reliable assay method for EPI compared with other technologies, when assessing the relationship between EPI concentrations and AD (Raum and Swerdloff, 1981).

Also, subgroup analysis indicated that NE concentrations were lower in AD patients than in the controls for subjects aged at least 80. Previous studies proposed that age-associated NE deficiency is implicated in the pathophysiology of AD. For instance, some animal studies demonstrated that NE can treat and relieve the oxidative stress of aging rats cells (Schraml et al., 2007). Moreover, there was a correlation between a decrease in beta-adrenergic receptor-mediated neuromodulatory actions and NE, which contributed to the age-related memory declines (Bickford, 1995). Some clinical studies also suggested that aging-related emotional memory deficits could be reversed by NE via regulating the stability of surface AMPA receptors (Luo et al., 2015). These findings, therefore, are consistent with the association between NE and AD found in this study among subjects with at least 80 years of age, implying that NE may play a pivotal role in the pathophysiology of AD; hence, it may be a potential candidate for treating aging-related memory deficits.

To date, a number of factors have been proposed to cause age-induced damage to the brain, including oxidative stress, free radical damage, and intracellular fibrillary tangles, which can be modified by aging, and have been associated with occurrence of AD (Mohsenzadegan and Mirshafiey, 2012; Tatsumi et al., 2014). Moreover, DA deficiency was found to contribute to age-related changes (Aliev et al., 2014). Meanwhile, previous research showed that DA density progressively reduced with aging and more severely in cases with AD (Rieckmann et al., 2018). Therefore, future clinical trials are needed to verify the potential therapeutic effectiveness of dopaminergic drugs in AD, particularly for AD patients aged 80 years or older.

Furthermore, the important neurotransmitters, catecholamines, are produced mainly in the sympathetic nervous system, adrenal medulla, and the brain (Ounissi et al., 2015). Therefore, after catecholamine synthesis, they are conserved in postganglionic neurons in membrane-bound storage vesicles within the chromaticity globules of the adrenal medulla and released into the circulation (Ounissi et al., 2015). Brain tissue or cerebrospinal fluid (CSF) catecholamine samples have a high degree of correlation with brain aging and progression of AD (Tank and Lee, 2015; Zhang and Gong, 2016). However, these two samples were difficult to obtain, so plasma was selected for most of the studies. Thus, previous studies have shown that plasma catecholamine analysis technology and stability have been extensively studied (Peaston and Weinkove, 2004). Detailed steps of collection and treatment of plasma catecholamines are described in the previous studies. For example, centrifugal heparinized blood is sufficient within 30 min of collection. Once plasma is isolated from blood cells, catecholamines can be stored for up to 1 year at −70°C (Peaston and Weinkove, 2004). However, plasma catecholamine samples are affected by diurnal variation, although 24-h urinary catecholamines circumvent this variation. Noteworthy, the stability of 24-h urinary catecholamines is still controversial; for example, catecholamine urine samples are prone to auto-oxidation at an alkaline pH (Grouzmann and Lamine, 2013). Overall, further research is needed to alert laboratory technicians of the possible pre-analysis problems of differences between different samples, including the standardization of the procedures for sample collection, transport, and storage conditions.

Additionally, contrary to EPI concentrations, significantly lower NE concentrations were found in AD patients than in the controls, when NE samples were assayed by HPLC, but no such difference was found between the two groups, when NE samples were assayed by other methods. Thus, compared with other assay technologies, this result may suggest that HPLC may be more reliable when investigating the relationship between NE concentrations and AD (Hows et al., 2004).

Furthermore, subgroup analysis indicated that females with AD had lower concentrations of NE than their counterparts, female controls. However, no such significant difference was apparent between the males with AD and male controls. It is well-known that females are at a greater risk of developing AD (Ferretti et al., 2018). Although the sex-specific clinicopathological AD phenotypes are largely unexplored, multiple factors have been suggested to underlie the observed association between AD and gender (Mosconi et al., 2017; Ferretti et al., 2018). Several lines of evidence support the gender-mediated genetic and endocrine hypothesis of AD. For example, multimodality brain imaging indicated sex differences in the development of AD. Also, it indicated that endocrine (progesterone and estrogen) changes play an important role in the process of female preclinical AD phase, which occurs during the female early aging process and coincides with the endocrine transition of perimenopause (Mosconi et al., 2017). Moreover, experimental studies demonstrated that NE neurons within the nucleus tractus solitarii and ventrolateral medulla represent gonadal steroid-dependent components of several neural networks regulating estrogen and progesterone (Haywood et al., 1999). In particular, estrogen modulated by NE can prevent memory impairment in AD (Martinez-Morales et al., 2001). Other clinical studies also observed low plasma NE concentrations in the elderly female with AD (Umegaki et al., 2000). Therefore, these previous findings are consistent with the results of this study that NE concentrations differed between the AD patients and controls with respect to the female gender. Conversely, male AD patients showed a significant decrease in EPI concentrations when compared with male controls, but these concentrations did not significantly differ between females with AD and female controls. Nevertheless, considering the small sample size of the male group, this result may not be reliable. Therefore, more studies are needed in the future to explore the role of EPI concentrations in the pathophysiological process of AD in males and females. Additionally, with respect to DA, subgroup analyses indicated that females with AD had lower concentrations of DA than their counterparts, female controls. In spite of that, no such significant difference was apparent between males with AD and male controls. Current studies of individuals with AD provide evidence of alterations in the neuroendocrine system that dopamine and acetylcholine are affected by sex steroid hormones (Giacobini and Pepeu, 2018). One explanation could be that sex hormones exert trophic effects on the cholinergic system, while acetylcholine is involved in dopaminergic mediators, which are responsible for progressive synaptic disarrangement, impairment of neurotransmission, and cell loss. Thus, this plays a key role in brain damage and aging and may explain why females are more vulnerable than males to the development of AD symptoms, which aggravate after perimenopause. Therefore, addressing gender differences in AD would be crucial for the development of precise and effective therapeutics for AD. In summary, gender-related differences in neural anatomy and function are starting to emerge, and gender might constitute an important factor for AD patients' stratification and personalized treatment.

There are some limitations to consider when interpreting the results of this review. First, the number of included participants in the eligible studies was relatively small. Next, all the eligible studies for this meta-analysis were cross-sectional studies; therefore, causal relationship or association could not be established. Last, the present study did not consider confounders, which may affect concentrations of catecholamines, such as blood pressure, smoking, body mass index, alcohol drinking status, and physical activity (Walker and Kane, 2002; Saxena et al., 2014). That is, the majority of the eligible studies for this meta-analysis did not report these confounders. Therefore, it is necessary that future studies on this subject should take the preceding confounders into consideration.

## Conclusion

This meta-analysis suggests that AD was associated with decreased concentrations of DA and NE, but not with the changes in EPI concentrations. Although the molecular underpinnings of the decrease of DA and NE in AD remain to be confirmed, the foregoing results might open up new perspectives in the early diagnosis, identification of novel neuroimaging biomarkers, and provision of novel targets for pharmacological interventions. Nevertheless, further research is needed to ascertain whether NE could be used as a diagnostic tool.

## Data Availability Statement

All datasets generated for this study are included in the article/Supplementary Material.

## Author Contributions

AL and XP participated in study design. XP, PJ, KA, and AK contributed to the analysis, quality assessment data collection, statistical analyses and interpretation of results, and drafting and revising the manuscript. SW participated in the critical revision discussion and manuscript revision. All authors contributed to the article and approved the submitted version.

## Conflict of Interest

The authors declare that the research was conducted in the absence of any commercial or financial relationships that could be construed as a potential conflict of interest.
