# Supplementary material for: Catecholamines in Alzheimer's Disease: A Systematic Review and Meta-Analysis
Source: Front Aging Neurosci. 2020 Sep 11;12:184. doi: 10.3389/fnagi.2020.00184 (PMC7516036; doi:10.3389/fnagi.2020.00184)
Supplement: Supplementary file 1 [file Table_1.doc]

**Search strategies: details of search strategy.**

((Alzheimer's Disease[Title] OR Alzheimer Syndrome[Title] OR Alzheimer Dementia[Title] OR Alzheimer[Title] OR AD[Title])) AND (Catecholamine[Title] OR Catecholamines[Title] OR Dopamine[Title] OR Dopamin[Title] OR Epinephrine[Title] OR Norepinephrine[Title] OR Noradrenaline[Title] OR Noradrenalin[Title] OR Adrenaline[Title] OR Adrenalin[Title])

144 of PubMed

(TITLE: (((((((((Catecholamine OR Catecholamines) OR Dopamine) OR dopamine) OR Epinephrine) OR Norepinephrine) OR Noradrenaline) OR Noradrenalin) OR Adrenaline) OR Adrenalin) AND TITLE: ((((Alzheimer's Disease OR Alzheimer Syndrome) OR Alzheimer Dementia) OR Alzheimer) OR AD))

231 of Web of Science

('catecholamine':ti OR 'catecholamines':ti OR 'dopamine':ti OR 'dopamin':ti OR 'epinephrine':ti OR 'norepinephrine':ti OR 'noradrenaline':ti OR 'noradrenalin':ti OR 'adrenaline':ti OR 'adrenalin':ti) AND ('alzheimers disease':ti OR 'alzheimer syndrome':ti OR 'alzheimer dementia':ti OR 'alzheimer':ti OR 'ad':ti)

184 of Embase

TI ( Alzheimer's Disease OR Alzheimer Syndrome OR Alzheimer Dementia OR Alzheimer OR AD ) AND TX ( Catecholamine OR Catecholamines OR Dopamine OR Dopamin OR Epinephrine OR Norepinephrine OR Noradrenaline OR Noradrenalin OR Adrenaline OR Adrenalin )

148 of PsycARTICLES
